# Accommodative Exercises to Lower Intraocular Pressure

**DOI:** 10.1155/2020/6613066

**Published:** 2020-12-18

**Authors:** Thomas J. Stokkermans, Jeremy C. Reitinger, George Tye, Chiu-Yen Kao, Sangeetha Ragupathy, Huachun A. Wang, Carol B. Toris

**Affiliations:** ^1^University Hospitals Cleveland Medical Center, Cleveland, Ohio, USA; ^2^Case Western Reserve University, Cleveland, Ohio, USA; ^3^Creighton University School of Medicine, Omaha, Nebraska, USA; ^4^Claremont McKenna College, Claremont, California, USA; ^5^University of Nebraska Medical Center, Omaha, Nebraska, USA

## Abstract

**Purpose:**

This study investigated how a conscious change in ocular accommodation affects intraocular pressure (IOP) and ocular biometrics in healthy adult volunteers of different ages.

**Methods:**

Thirty-five healthy volunteers without ocular disease or past ocular surgery, and with refractive error between −3.50 and +2.50 diopters, were stratified into 20, 40, and 60 year old (y.o.) age groups. Baseline measurements of central cornea thickness, anterior chamber depth, anterior chamber angle, cornea diameter, pupil size, and ciliary muscle thickness were made by autorefraction and optical coherence tomography (OCT), while IOP was measured by pneumotonometry. Each subject's right eye focused on a target 40 cm away. Three different tests were performed in random order: (1) 10 minutes of nonaccommodation (gazing at the target through lenses that allowed clear vision without accommodating), (2) 10 minutes of accommodation (addition of a minus 3 diopter lens), and (3) 10 minutes of alternating between accommodation and nonaccommodation (1-minute intervals). IOP was measured immediately after each test. A 20-minute rest period was provided between tests. Data from 31 subjects were included in the study. ANOVA and paired *t*-tests were used for statistical analyses.

**Results:**

Following alternating accommodation, IOP decreased by 0.7 mmHg in the right eye when all age groups were combined (*p* = 0.029). Accommodation or nonaccommodation alone did not decrease IOP. Compared to the 20 y.o. group, the 60 y.o. group had a thicker ciliary muscle within 75 *μ*m of the scleral spur, a thinner ciliary muscle at 125–300 *μ*m from the scleral spur, narrower anterior chamber angles, shallower anterior chambers, and smaller pupils during accommodation and nonaccommodation (*p*'s < 0.01).

**Conclusion:**

Alternating accommodation, but not constant accommodation, significantly decreased IOP. This effect was not lost with aging despite physical changes to the aging eye. A greater accommodative workload and/or longer test period may improve the effect.

## 1. Introduction

Currently, slowing of glaucoma progression is accomplished primarily by management of intraocular pressure (IOP) through pharmacological and surgical methods. Outside of diabetic control and following healthy lifestyle choices [1], there is a dearth of modifiable behaviors to decrease IOP in patients with ocular hypertension or glaucoma. Conscious accommodation has shown some promise as a potential at-home exercise to lower IOP [2–7]. Yet, accommodative effort only reduces IOP by a small amount. Finding a way to maximize and sustain this reduction in IOP is still needed to approach healthy levels that are of clinical significance [2, 6].

The drainage of aqueous humor from the eye changes according to accommodative state: primarily occurring through the uveoscleral pathway during nonaccommodation and through the trabecular pathway during accommodation [8]. One question that has not been sufficiently explored is whether alternating between accommodative states and thus aqueous-humor outflow pathways could lead to a more significant decrease in IOP than sustained accommodation alone. In theory, nonaccommodation causes relaxation of the ciliary muscle allowing a greater flow of aqueous humor into the muscle and downstream. At the same time, the ciliary muscle is not pulling on the scleral spur and aqueous-humor flow through the trabecular outflow pathway is slow at best. During accommodation, the ciliary muscle contracts and eliminates the spaces between its muscle bundles. The aqueous humor within the muscle is squeezed out and new aqueous humor cannot flow in, thus slowing uveoscleral outflow. Simultaneously, the ciliary muscle pulls the scleral spur posteriorly, increases the area within Schlemm's canal, and reduces resistance to trabecular outflow [8, 9]. Trabecular outflow more than offsets the decrease in the uveoscleral outflow during accommodation and a decrease in IOP ensues [10]. During alternating accommodation, we hypothesize that ciliary muscle contraction and relaxation alternates aqueous humor flow between the two outflow pathways, improving total outflow and reducing IOP.

The only prior study to consider this hypothesis had asked subjects to alternate between accommodation and nonaccommodation every 3 minutes [4]. That study found a 16% greater decrease in IOP with alternating accommodation than accommodation alone, but the difference was not significant at *p* = 0.35 [4]. The rationale to alternate every 3 minutes was based on a 1961 study [2] that found a maximum IOP reduction after 2.7 minutes (for ages 20–25) and 4.0 minutes (for ages 45–55) of sustained accommodation. However, the same study showed a reduction in IOP as early as 1 minute [2]. In a later study [5], a maximum reduction in IOP was found within the first 30 seconds of sustained accommodation. The goal of the current study is to assess whether a 1-minute cycle of alternating accommodation states would be more effective than sustained accommodation alone. If successful, these findings could be utilized to develop conscious eye exercises to lower IOP.

## 2. Methods

### 2.1. Subject Recruitment and Screening

The study was approved by the Institutional Review Board at University Hospitals Cleveland Medical Center and performed in accordance with the Declaration of Helsinki. Informed consent was obtained prior to initiating any study-related procedures. Subjects enrolled in the study were healthy volunteers in three age groups: 20–25 y.o., 40–49 y.o., and 60–69 y.o. Exclusion criteria included current use of ocular medications, past history of dry eye, ocular inflammation, ocular surgery, and amblyopia, as well as a spherical equivalent refractive error of greater than minus 3.5 or plus 2.5 diopters of either eye. Use of systemic medications was not an exclusion criterion because each subject served as his/her own control [11]. All subjects received a comprehensive eye examination within one month prior to enrolling in the study.

### 2.2. Experimental Design

The effects of accommodation, nonaccommodation, and alternating accommodation on IOP were tested using a randomized, crossover study design. Given the nature of the study, the subjects and investigators were not masked to the testing conditions or age of the subject. However, the ciliary muscle images were masked to the investigator evaluating images. Baseline measurements of both eyes taken just before the first test included pupil size (Grand Seiko WAM-5500 Binocular Autorefractor/Keratometer, AIT Industries, Bensenville, IL) and anterior chamber depth, cornea thickness, and anterior chamber angle (Visante Cirrus OCT, Carl Zeiss Meditec Inc., Dublin, CA). Thickness of the temporal ciliary muscle of the left eye was measured from OCT images of the ciliary muscle. For all exercises, the left eye was directed toward a black screen. The right eye focused on an iPhone at a distance of 40 cm. The iPhone displayed an e-book with a standard text size (20/40 or 0.3 logMar) (20 and 40 y.o. groups) or a cartoon for the 60 y.o. group, as the presbyopia in this group made it difficult to read standard text size. A lens was placed at the standard vertex distance of 12 mm that corrected for refractive error. An additional +2.5 diopter lens was added to allow for a clear view of the target without accommodation. For the accommodation test, a minus 3 diopter lens was added to stimulate accommodation without convergence.

**Figure 1:**
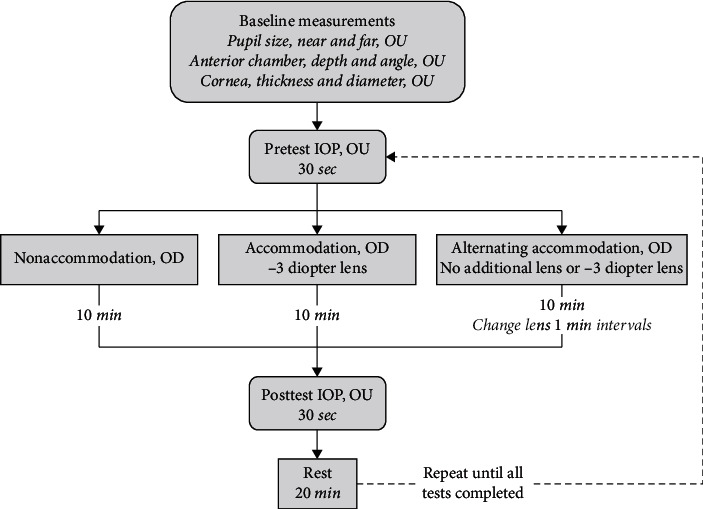
Study design and measurements.

Each subject underwent three tests in random order: (1) 10 minutes viewing the target with no accommodation (nonaccommodation), (2) 10 minutes viewing the target through a −3 diopter lens (accommodation), and (3) 10 minutes viewing the target while alternating between −3 and 0 diopters of accommodation power at 1 minute intervals (alternating accommodation). To stabilize the head during each test, subjects were asked to keep their chin on the chinrest of the OCT instrument. Head and eye movements were watched on the OCT monitor by the investigator. During the tests, subjects were coached to focus to the best of their ability, keep their head still on the chinrest and not shift their gaze away from the target. IOP measurements were made within 2 minutes following the completion of each test. The subjects were given a 20-minute rest period between each test, during which time they were instructed to walk or nap and avoid any activity requiring near vision. After returning from their rest period, the subjects repeated IOP measurements before beginning the next test. A summary of study procedures is found in Figure 1.

### 2.3. Experimental Measurements

IOP measurements of both eyes were taken with a pneumatonometer (Classic model 30, Reichert, DePew, NY) after topical anesthesia with proparacaine hydrochloride ophthalmic solution USP, 0.5% (Bausch Health Companies Inc., Laval, Quebec, Canada). Two measurements were obtained and if the values were more than 3 mmHg apart, a third measurement was made. The IOPs within 3 mmHg of each other were averaged. Data were saved on a computer using LabChart software (AD Instruments, Colorado Springs, CO). IOPs were collected at baseline and within 2 minutes following completion of each test, for a total of six sets of measurements.

OCT images of the anterior segment were taken of the left eye at baseline in accordance with the instrument protocol. The subject sat in front of the OCT facing the camera lens and looked through the eyepiece. From these images, measurements were made of the cornea thickness, anterior chamber depth, anterior chamber angle, and corneal diameter. Next, the subject rotated his/her head to the right so that the right eye could see a distant target on the wall 10 feet away and the temporal ciliary muscle of the left eye was clearly visible on the OCT screen. Two to three images of the ciliary muscle of the left eye were collected. The ciliary muscle images were sent to one investigator (CYK) who used a customized computer program to measure the ciliary muscle thickness starting at 25 *µ*m from the scleral spur and in 25 *μ*m increments up to 300 *μ*m from the scleral spur [12, 13]. The investigator was masked to the subject's age. Once all measurements were entered into a spreadsheet, the images were unmasked and the data were separated by the age groups.

### 2.4. Data Analysis

The effects of age and accommodation state on the pupil size and anterior chamber (AC) measurements were analysed using one-way ANOVA models with Turkey post hoc HSD. Multiple imputation was used for missing pupil size data. The effects of age on change in IOP from baseline were analysed using a paired *t*-test and a type-III sums of squares two-way ANOVA. The effects of accommodation state on change in IOP were analysed using a paired *t*-test and one-way ANOVA. The effects of age on ciliary muscle thickness were analysed using a linear mixed-effects model. Statistical tests were done with *R* software (RStudio, Boston, MA). Statistical significance for all tests was presumed if *p* < 0.05. Data are presented as means ± standard deviation.

## 3. Results

### 3.1. Subjects

Thirty-five subjects were eligible for the study following the screening exam. One subject withdrew during the study after experiencing corneal discomfort. Data from the first three subjects were omitted after changes in study design were made to optimize the timing of test periods and physical layout of the lens and OCT imaging. Thirty-one subjects completed the study with the new study design (Table 1). Baseline IOP values in right and left eyes were similar for all age groups (Table 2).

### 3.2. Intraocular Pressure

**Figure 2:**
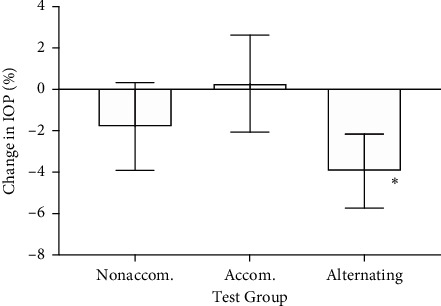
Percent change in IOP from pretest, right eye. Nonaccom, focusing without accommodation on target for 10 minutes; Accom, focusing on target through −3 diopter lens for 10 minutes; Alternating, alternating between nonaccommodation and accommodating conditions every minute for 10 minutes. Asterisk indicates *p* < 0.05 when compared to baseline, paired *t*-test. Error bars indicate standard error.

When compared to the pretest IOP, there was a 0.7 mmHg decrease (−3.9%) in IOP of the right eye during alternating accommodation when all age groups were combined (Figure 2). This difference was significant when using a paired *t*-test at *p* = 0.029, but not when using a one-way ANOVA. Continuous accommodation did not decrease IOP (*p* = 0.65). When examining the 60 y.o. group specifically, there was a 1.6 mmHg decrease in right-eye IOP during the nonaccommodation test (*p* < 0.01) and a 1.1 mmHg decrease in right-eye IOP during the alternating accommodation test (*p* = 0.028). However, the statistical significance of these effects was lost when tested by the two-way ANOVA.

Compared to baseline, there was a decrease in right-eye IOP after each test, showing significance at *p* = 0.02 for nonaccommodation, *p* = 0.02 for accommodation, and *p* = 0.01 for alternating accommodation. A reduction in IOP with repeated measurements is frequently reported in the literature, which reinforced the need for test randomization. IOPs returned to baseline levels after each 20-minute rest period.

The IOP changes found in the right eye were not found in the left eye: there were no significant differences across age groups or tests with either a paired *t*-test or ANOVA. The difference in IOP change between right and left eyes was significant at *p* < 0.01. There was a +4% change in IOP during nonaccommodation for the left eye (vs. −1.8% for the right eye) and a +1.1% change in IOP during alternating accommodation for the left eye (vs. −4% for the right eye).

### 3.3. Biometrics

**Figure 3:**
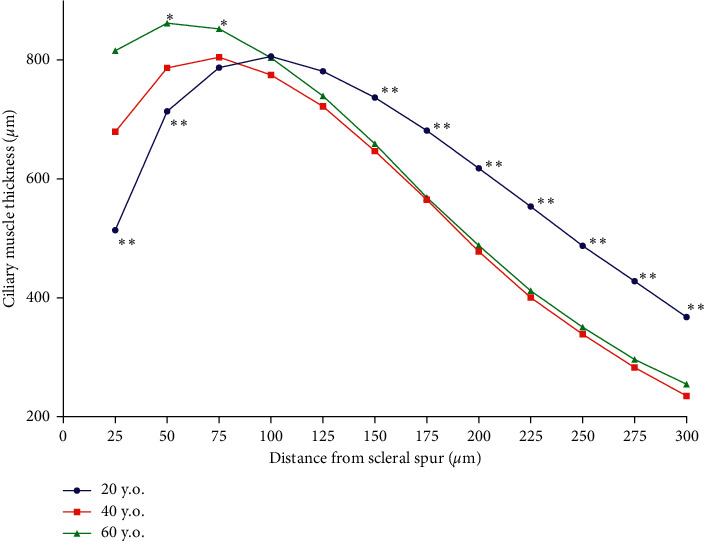
Ciliary muscle thickness in age groups of 20 y.o., 40 y.o., and 60 y.o. while nonaccommodating. The ciliary muscle of the 60 y.o. group was thicker within 75 *μ*m of the scleral spur compared to the 20 y.o. and 40 y.o. groups. The ciliary muscle of the 20 y.o. group was thinner up to 50 *μ*m from the scleral spur and thicker at distances of 125–300 *μ*m from the scleral spur compared to the 40 y.o and 60 y.o. groups (^*∗∗*^*p* < 0.00001), using linear mixed-effects models (^*∗*^*p* < 0.00001).

Compared to the 20 y.o. group, the 60 y.o. group had a thicker ciliary muscle within 75 *μ*m of the scleral spur (*p* < 0.001) and thinner ciliary muscle at a distance of 125–300 *μ*m from the scleral spur (*p* < 0.01) (Figure 3). Compared to the 20 y.o. group, the 60 y.o. group had narrower anterior chamber angles (36.4 ± 7.1 vs 47.6 ± 6.0 degrees, respectively; *p* = 0.001) and shallower anterior chambers (2.94 ± 0.42 vs 3.22 ± 0.24 mm, respectively; *p* = 0.001) (Table 2). At baseline, the 20 y.o. group had larger pupils than both the 40 y.o. and the 60 y.o. groups (*p* < 0.01), while the 40 y.o. and 60 y.o. groups had similar pupil sizes (*p* > 0.05) (Table 2).

## 4. Discussion

This study tested the idea of lowering IOP by consciously alternating accommodation through eye exercises. IOP was slightly reduced during the alternating accommodation test, as predicted. Our findings showed that sustained accommodation did not lower IOP. Other studies investigated this also with varying results. The method of accommodation was different among the studies and may explain the different findings. In studies reporting that sustained accommodation reduces IOP, subjects focused on objects at far and near distances to induce accommodation [3, 4, 6, 7]. However, two studies found that accommodation produced by lenses had no effect on IOP of emmetropic subjects and, in fact, increased IOP in myopic subjects [14, 15]. Apparently, lens-induced accommodation may not recruit miosis within the near response triad (accommodation, convergence, and miosis) [6, 8]. There has been long-standing controversy over which stimuli, retinal disparity (resulting in vergence), or blur (resulting in accommodation) activates the pupillary near response. Accommodation without convergence did not induce pupillary constriction in some studies [16–18] while pupillary changes were noted in other studies [19–21]. The most current understanding involves a complex pathway of neural controllers for both vergence and accommodation, and that neither neural controller works alone to activate the pupillary near reflex [8, 19, 22]. Vergence causes a greater pupillary change than blur-driven accommodation alone [18, 19]. Absence of vergence in our study may have limited the magnitude of IOP response. Pupil changes were observed while watching the subject's left eye on the OCT monitor but the pupil size could not be accurately measured during the accommodative tests.

Our finding that the IOP of the right eye (focusing on target) was lower than the IOP of the left eye (viewing black screen) during the alternating-accommodation test is unexpected and intriguing. A couple of explanations for this phenomenon could be possible. (1) Lateral gaze might differentially affect IOP of each eye as has been reported previously [23]. In the published study, more extreme lateral gaze was examined with the finding that IOP was lower in the eye directed nasally [23]. An explanation is that tension on the sclera might be opening up the supraciliary and suprachoroidal spaces and improving the uveoscleral drainage, which in turn lowers IOP. While attempts were made to eliminate lateral gaze in the current study, a small degree of right lateral gaze was present. (2) Although it is presumed that the accommodative reflex is symmetric because of its bilateral neural pathway [8], the accommodative demand placed on each eye is known to be asymmetrical during lateral gaze and anisometropia (corrected or uncorrected) [24–26]. Controversy exists over whether the eye has the ability to respond to this discrepancy with asymmetric accommodative power, termed aniso-accommodation. When presented with an asymmetric accommodative stimulus, some studies have found an equal accommodative response between eyes [27, 28], while others have found a difference in accommodative power between eyes of up to 25% after a training period [29, 30]. It is also known that aniso-accommodation is prevalent in anisometropic amblyopia [31, 32]. Within our study, the difference in accommodative demand between the right and left eye could have been responsible for the lack of change in IOP within the reflexively accommodating eye (left).

A few study limitations should be mentioned. This was a pilot study with a small number of subjects and small changes in IOP. Nevertheless, it does answer some design questions that could be implemented in a larger study to obtain more robust results. Another limitation was that the shape of the OCT instrument prevented imaging the ciliary muscle of the eye that was actively focusing on a target during the test. The final limitation of this study was that, with the instruments available to us, we could not monitor the extent to which each subject was attempting to focus or if they were focusing with equal effort during the whole test. However, we did monitor head and eye movements and coached the subjects to make every effort to focus. The actual effort made by the subject to focus could have resulted in variation in both the amount and duration of accommodative power of each subject.

In summary, our findings indicate that repeated eye accommodation was more effective at lowering IOP than sustained accommodation alone. An improvement in IOP lowering might be possible with an increase in frequency of accommodative changes, a longer period of exercise, and/or a stronger accommodative workload. Repeated accommodation eye exercises could be used by patients as an at-home intervention for lowering IOP. Future studies should directly compare IOP reduction according to the mode of accommodation (lens vs distance-induced) and laterality of accommodation (unilateral vs bilateral stimulus). Additionally, future studies should directly compare the amount of contraction of the ciliary muscle during active and reflexive accommodation.

## Figures and Tables

**Table 1:** Demographic characteristics of study participants.

	Young age, *n* = 11	Middle age, *n* = 9	Old age, *n* = 11
Age, years old	23.5 ± 1.6	45.2 ± 3.4	62.9 ± 2.3
Sex, male/female	2/9	4/5	4/7
Ethnicity, Asian/black/white	2/1/8	1/5/3	0/7/4

**Table 2:** Ocular characteristics of study participants.

	Young age *n* = 11	Middle age *n* = 9	Old age *n* = 11	*p* ^*∗*^
Baseline IOP, OD mmHg	16.99 ± 2.91	20.10 ± 3.39	19.21 ± 3.17	0.09
Baseline IOP, OS mmHg	17.32 ± 2.2	19.78 ± 3.10	19.22 ± 3.75	0.18
CCT, mm	0.57 ± 0.03	0.56 ± 0.03	0.54 ± 0.03	0.12
AC depth, mm	3.22 ± 0.33	2.92 ± 0.42	2.67 ± 0.31	<0.01
AC diameter, mm	12.08 ± 0.55	12.05 ± 0.25	11.94 ± 0.33	0.72
AC angle, right, deg	47.60 ± 6.00	38.7 ± 7.08	36.4 ± 7.12	<0.01
AC angle, left, deg	44.79 ± 7.49	36.89 ± 9.33	32.5 ± 7.34	<0.01
Near pupil, OD, mm	6.75 ± 0.79	4.41 ± 0.77	4.45 ± 0.84	<0.0001
Near pupil, OS, mm	5.88 ± 0.92	4.03 ± 0.58	4.34 ± 0.73	<0.0001
Far pupil, OD, mm	6.98 ± 0.68	4.98 ± 0.85	4.86 ± 0.83	<0.0001
Far pupil, OS, mm	6.43 ± 1.05	4.80 ± 0.97	4.71 ± 0.66	<0.01

CCT, central cornea thickness; AC, anterior chamber depth; near pupil, pupil size during accommodation; far pupil, pupil size during nonaccommodation. *p* value was obtained by the one-way ANOVA comparing all three age groups.

## Data Availability

The data used to support the findings of the study are available upon request to the corresponding author.
